# Analytical and Experimental Investigation of a Curved Piezoelectric Energy Harvester

**DOI:** 10.3390/s22062207

**Published:** 2022-03-12

**Authors:** Talieh Pourashraf, Philip Bonello, Jason Truong

**Affiliations:** Department of Mechanical, Aerospace and Civil Engineering, University of Manchester, Manchester M13 9PL, UK; philip.bonello@manchester.ac.uk (P.B.); jason_truong@hotmail.com (J.T.)

**Keywords:** energy harvesting, curvature, dynamic stiffness method, piezoelectric, ANSYS

## Abstract

Piezoelectric energy harvesters have traditionally taken the form of base excited cantilevers. However, there is a growing body of research into the use of curved piezoelectric transducers for energy harvesting. The novel contribution of this paper is an analytical model of a piezoelectric energy harvesting curved beam based on the dynamic stiffness method (DSM) and its application to predict the measured output of a novel design of energy harvester that uses commercial curved transducers (THUNDER TH-7R). The DSM predictions are also verified against results from commercial finite element (FE) software. The validated results illustrate the resonance shift and shunt damping arising from the electrical effect. The magnitude, phase, Nyquist plots, and resonance frequency shift estimates from DSM and FE are all in satisfactory agreement. However, DSM has the advantage of having significantly fewer elements and is sufficiently accurate for commercial curved transducers used in applications where beam-like vibration is the predominant mode of vibration.

## 1. Introduction

Vibration energy harvesting involves the scavenging of ambient kinetic energy by transforming it to electrical energy using electromagnetic, electrostatic or piezoelectric devices [1,2,3,4,5]. Due to their effectiveness, light weight and small size, piezoelectric devices are extensively used as an alternative to batteries in low power devices [6]. Piezo materials can be integrated into lightweight structures, such as beams and plates, to convert electrical power into mechanical energy (actuator mode) or mechanical power into electrical energy (sensor mode or vibration energy harvesting mode). Considerable research into the modelling and analysis of piezoelectric devices has been performed with regard to actuators and sensors for smart vibration control, e.g., [7,8], vibration energy harvesting, e.g., [9,10,11,12,13,14,15,16], or even simultaneous vibration control and vibration energy harvesting, e.g., [17,18]. The focus of the present paper is vibration energy harvesting.

A review of vibration energy harvesting literature [19] shows a number of publications researching configurations designed to access the maximum voltage via various mechanisms, e.g., induced by moving loads on civil structure applications [12], vehicles travelling on a bridge [20], and aeroelastic galloping [21]. Sodano et al. [13] conducted an experimental comparison of the power generation capabilities of a variety of piezoelectric materials. With regard to mathematical modelling techniques, although numerical methods were used as early as 2002 [14], significant progress on modelling the electromechanical coupling was made from 2008 onwards following the analytical approach of Erturk and Inman [9,11,22,23].

Erturk and Inman [9] utilized the Euler–Bernoulli beam theory along with the constitutive equations of piezoelectric material to set up a distributed parameter model of a base-excited energy harvesting cantilever made up of a unimorph (i.e., single piezoelectric layer bonded to a metallic substrate). This model was then transformed using a modal transformation wherein the clamped-free modes with no electrical effect (short circuit conditions) were used to represent the flexural vibration of the beam relative to the vibrating base with a resistive shunt circuit. The resulting equation of motion (considering only one mode) was solved to obtain the displacement, voltage, and power response for harmonic excitation over a range of frequencies. The same authors later performed a similar analysis of base-excited cantilevered bimorph with tip mass, which was additionally validated by experimental investigation [9]. Both [9,22] illustrate the vibration damping effect and shift in response frequency induced by the electrical coupling.

Rafique and Bonello [24] also used the above-described analytical modal analysis method (AMAM) of distributed parameter energy harvested cantilevers to model a bimorph, this time without tip mass, and obtained satisfactory correlation with experimental results for voltage, displacement, and power frequency response functions (FRFs). For a more thorough validation, Nyquist plots of the FRFs were considered in addition to the more usual magnitude FRF plots. In [25], Dalzell and Bonello extended the AMAM approach to an experimentally validated analysis of an energy harvesting cantilevered bimorph shunted by an energy storage circuit comprising a diode in series with a capacitor for half-wave AC/DC rectification.

In another study, Bonello and Rafique [26] introduced the dynamic stiffness method (DSM) for the analysis of base-excited cantilevered unimorph and bimorph devices with or without tip mass. The model was based on the Euler–Bernoulli beam model with piezoelectric coupling and its results were verified against those obtained with the AMAM. The DSM can be extended beyond simple uniform-section cantilever beams more readily than AMAM. It was shown in [26] that the DSM could be applied to more complex systems composed of an assembly of straight piezoelectric beam segments with various boundary conditions using matrix assembly techniques similar to those used in the finite element (FE) method. However, unlike FE or AMAM, the DSM uses the exact function to describe the segment’s vibrating shape under harmonic excitation. Hence, for the purpose of frequency domain analysis, the DSM offers an efficient computational approach in which the accuracy is independent of element size, unlike FE [27,28].

In most of the aforementioned research, the energy harvesting devices have taken the typical form of base-excited cantilevered straight beams with/without a tip mass. However, there is a growing body of research into the use of curved piezoelectric transducers for energy harvesting purposes. Such curved transducers were originally designed as actuators, and compared to their traditional counterparts, these curved devices not only offer more flexibility and reliability, but are also capable of being applied to harvest energy from low-frequency vibrating structures [10]. Examples of curved piezoelectric transducers that have been researched are RAINBOW [29], LIPCA [30], and THUNDER [10,31], with the latter being considered the most promising due to its unique performance features compared to traditional unimorph, bimorph, and straight extensional actuators [32]. THUNDER (thin layer unimorph ferroelectric driver) consists of stainless-steel substrate, the piezo-electric wafer, and an aluminum top layer and adhesive bonding layers. Its curved shape is the result of prestress induced during the manufacturing process of the device. After processing in an autoclave, a curved prestressed device with high out-of-plane displacement is formed, in part due to a mismatch between the coefficients of thermal expansion corresponding to the PZT and the metal layer [32,33]. Mossi et al. [34] showed that THUNDER devices’ performances can be significantly affected by design parameters, such as the geometry, number of layers, and the thickness of the layers. The same conclusion for other types of THUNDER were also observed in [35].

In 2016, Wang et al. [31] investigated how to maximize energy harvesting efficiency by changing the radius of curvature of a THUNDER as a tuning parameter. The first part of their study involved an experimental and analytical investigation for the output voltage and power across a load considering both standard AC and full wave AC/DC rectification. The thin shell device was modelled as a curved laminated beam and it was further assumed that the curvature was sufficiently low so that the curvilinear coordinate along the neutral surface could be used interchangeably with the rectangular coordinate. Moreover, the analysis represented the vibrating shape using a modal transformation approach based on only one mode (i.e., a Rayleigh–Ritz procedure). The effect of changing the radius of curvature of the THUNDER was investigated in the second part of [31] but no experimental validation was provided.

In 2017, Bharat Kathpalia et al. [10] studied, analytically and experimentally, the implementation of a commercial arched piezo energy harvester (precisely a THUNDER configuration) for smart paver tiles. Closed-form expressions for the voltage output to force input frequency response function (FRF) and power output to force input FRFs, for a simple resistive load, were derived and experimentally validated for range low-frequency (<10 Hz). It was concluded that tens of microwatts of power could be produced by a single arch at low force levels, and milliwatt levels of power can be achieved by using several arches.

In 2018, Hasan et al. [36] established an FE method to address the analysis of piezoelectric coupled fields. The main idea behind this research was to study the effect of changing the radius of curvature to see how it changes the output voltage and power of the THUNDER device. They reported a notable agreement in experimental data and modal analysis carried out by ANSYS. A more recent study, carried out by Thonapalin et al. [37] in 2021, discussed the influence of temperature on different types of THUNDER samples, such as THUNDER 6R, 7R, and 8R. The experiments revealed, among other things, that high temperature badly affects the energy harvesting capability of the devices, particularly in the low frequency regime (e.g., reduction of 40% at 80 ℃) [37].

Motivated by the above-described advantages of the DSM, the novel contribution of the present paper is an analytical model of a piezoelectric energy harvesting curved beam based on the DSM and its application to predict the measured output of an energy harvester that uses commercial curved transducers (THUNDER TH-7R). The DSM predictions are also verified against results from commercial FE software. In the DSM approach the transducer is modelled as a curved laminated beam but, unlike [31] (where the Rayleigh–Ritz approach was used), no limitations on the degree of initial curvature are assumed. The analysis will include all layers of the transducer, including the adhesive layer, which was omitted in energy harvesting work [27,32] despite earlier evidence of its influence on actuation [29]. The energy harvesting device is designed such that the input excitation is applied to one end of the parallel transducers in the longitudinal direction, vibrating a tip mass attached at the other end. This design is novel for energy harvesting purposes, but a similar design has been used as an adaptive tuned vibration absorber by Bonello et al. [38] wherein the THUNDER devices were used in actuator mode to control the curvature and hence the tuned frequency.

## 2. Modelling by Dynamic Stiffness Method

**Figure 1:**
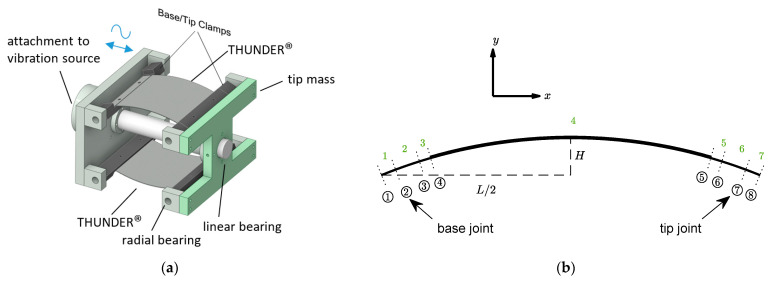
Vibration energy harvester: (**a**) 3D model of the assembly and (**b**) side view of one THUNDER device divided into five segments for analysis by DSM.

The linear constitutive law is incorporated into the equations of motion of a laminated curved beam with constant radius of curvature. Assuming harmonic vibrations, the dynamic stiffness method (DSM) is then applied to obtain the dynamic stiffness matrix of a curved piezoelectric beam. The DSM method is applied to the energy harvesting device in Figure 1, which consists of two THUNDER devices pinned together at both ends with a tip mass, whose electrodes are connected in parallel. Utilizing a matrix assembly process enables the inclusion of attachments to the curved beams, and boundary conditions.

The material properties and the dimensions of the THUNDER device are given in Table 1 and Table 2, respectively. The tip mass in Figure 1a is 410 g, including the mass of radial bearings at the free end.

### 2.1. Equations of a Curved Piezoelectric Beam Segment

Although THUNDER is actually a curved thin plate rather than a curved thin beam, the way the transducers are set up in the energy harvester design (Figure 1), allows each to be modelled as a curved thin beam with a good approximation.

**Figure 2:**
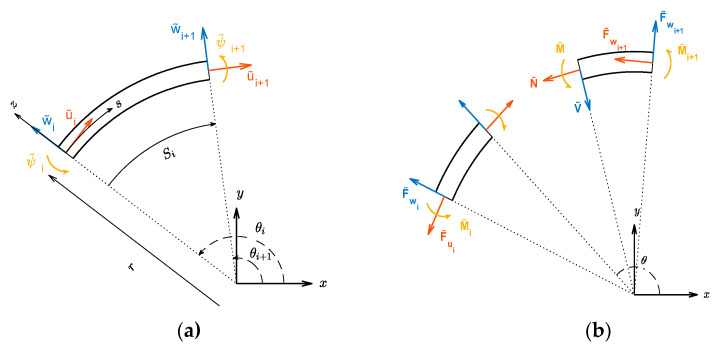
Dynamic stiffness analysis of segment of curved beam: (**a**) one element with nodal DOFs, local and global coordinate systems (**b**) element’s section with internal and external forces.

The strain for a curved thin beam is as follows.

(1) $$\varepsilon =\frac{\partial u}{\partial s}+\frac{w}{r}-z\frac{\partial^{2}w}{\partial s^{2}}+\frac{z}{r}\frac{\partial u}{\partial s}$$

where u and w are respectively the tangential and radial deformations at mid-surface, r is the radius of curvature at mid-surface, s is the curvilinear coordinate along the mid-surface, and *z* is the distance between the evaluation point and the mid-surface in the thickness direction (see local coordinate axes in Figure 2). Stress and charge density can be expressed as follows [39].

(2a) $$\sigma_{k}=Y_{k}\varepsilon \;\left(k\neq p\right),$$

(2b) $$\sigma_{p}=Y_{p}\varepsilon +e_{31}v/h_{p}$$

(3) $$D_{3,p}=e_{31}\varepsilon -\epsilon_{33}^{S}v/h_{p}$$

where subscript k refers to layer no. k (see Table 1), k=p refers to the piezo layer (as per Table 1, p=3), Y is the elastic modulus, h is the thickness, v is the voltage generated, $e_{31}$ is piezoelectric stress constant, and $\epsilon_{33}^{S}$ is permittivity at constant strain. To include material damping (*c*), the stress relations are modified as follows [26]:

(4a) $$\sigma_{k}=Y_{k}\varepsilon +c_{k}\dot{\varepsilon },$$

(4b) $$\sigma_{p}=Y_{p}\varepsilon +c_{p}\dot{\varepsilon }+e_{31}v/h_{p}$$

The equations of motions for a curved laminated beam in terms of displacements are given as:

(5a) $$K_{w}w+Ku+m\ddot{w}+\left({\bar{K}}_{w}{}_{a}+c_{a}\right)\dot{w}+\bar{K}\dot{u}+\chi_{w}v=0$$

(5b) $$-Kw+K_{u}u+m\ddot{u}-\bar{K}\dot{w}+\left({\bar{K}}_{u}+c_{a}\right)\dot{u}+\chi_{u}v=0$$

Note that $c_{a}$ represents the viscous damping coefficient and m is mass per unit length. The procedure to derive these equations and the expressions for constants (A, B, D) and operators ($K,\bar{K},$ and χ) are given in Appendix A.

For Rayleigh-type damping the following relations are used in advance (assuming the material damping coefficient distribution through thickness follows the same profile as modulus of elasticity).

(6a) $$\alpha =\frac{c_{a}}{m},$$

(6b) $$\;\;\beta =\frac{\bar{D}}{D}=\frac{\bar{B}}{B}=\frac{\bar{A}}{A}$$

where α and β are defined as mass proportional and stiffness proportional damping coefficients.

Following the same procedure applied in [40], α and β can be related to modal damping ratios $\zeta_{1}$ and $\zeta_{2}$ and frequencies $\omega_{1}$ and $\omega_{2}$ of the first two modes of vibration, resulting in the relations.

(7a) $$\alpha +\omega_{1}^{2}\beta =2\zeta_{1}\omega_{1}$$

(7b) $$\alpha +\omega_{2}^{2}\beta =2\zeta_{2}\omega_{2}$$

Since the experiments conducted (Section 4.2) cover only one mode (i.e., only $\zeta_{1}$ and $\omega_{1}$ are available) it is not possible to determine both coefficients α or β. In this case, one of the coefficients is arbitrarily set to zero and the other is fitted according to Equation (7a). In this paper α is set to zero (i.e., the stiffness proportional damping has been selected) and β is given by:

(8) $$\beta =2\zeta_{1}/\omega_{1}$$

Integrating the time rate of Equation (3) at midsection of piezoelectric layer ($z=z_{pc}$), and assuming that the electrode covers the extent of the segment (s=0, $s=S_{i}$) leads to electrical current equation:

(9a) $$I=b\int_{0}^{S_{i}}\left(e_{31}\dot{\varepsilon }-\epsilon_{33}^{S}/h_{p}\dot{v}\right)\;ds$$

(9b) $$\epsilon_{33}^{S}=\epsilon_{33}^{T}-\frac{e_{31}^{2}}{Y_{p}}$$

where $\epsilon_{33}^{T}$ is permittivity at constant stress. Substituting Equation (1) into Equation (9a) yields the subsequent formula for the current:

(10) $$I=-C_{p}\dot{v}+\dot{\Gamma }$$

where

(11a) $$C_{p}=\frac{S_{e}b\epsilon_{33}^{S}}{h_{p}}$$

(11b) $$\Gamma =be_{31}\left({u]}_{0}^{S_{i}}+\int_{0}^{S_{i}}\frac{w}{r}\;ds-z_{\mathrm{pc}}{\frac{\partial w}{\partial s}]}_{0}^{S_{i}}\right)$$

Notice that Equation (11b) neglects the last term of Equation (1) since z/r for a thin beam is negligible.

Assuming harmonic variation in time of the generated voltage with frequency ω rad/s (i.e., AC voltage) and an external load of generic impedance Z(ω), Equation (10) can be transformed into the frequency domain using complex notation as follows

(12) $$j\omega C_{p}\tilde{v}+\frac{\tilde{v}}{Z\left(\omega \right)}=j\omega \Gamma$$

where $\tilde{v}$ is the complex amplitude of the harmonic voltage v which is then given by v=Re{v˜ejωt} where Re{} denotes the real part of {} and $j=\sqrt{-1}$ denotes the imaginary unit.

### 2.2. Dynamic Stiffness Matrix of Curved Beam

Since the voltage is assumed to be harmonic, the vibration generating it is also harmonic, and the complex notation for the variables w and u in Equations (5) will be as follows

(13a) w(s,t)=Re{w˜(s)ejωt},

(13b) u(s,t)=Re{u˜(s)ejωt}

Substituting Equations (13) into Equations (5) and taking advantage of the separation of spatial and time variables in Equation (13), the homogenous part of the equations of motion will become as follows.

(14) $$\left[\begin{array}{cc} K_{w}-\omega^{2}m+j\omega \left({\bar{K}}_{w}+c_{a}\right) & K+j\omega \bar{K}\\ -\left(K+j\omega \bar{K}\right) & K_{u}-\omega^{2}m+j\omega \left({\bar{K}}_{u}+c_{a}\right) \end{array}\right]\left[\begin{array}{c} \tilde{w}\\ \tilde{u} \end{array}\right]=0$$

Now, assuming $\tilde{w}=ae^{\lambda s},\;\tilde{u}=\gamma ae^{\lambda s}$ and substituting in Equation (14), the eigenvalue problem is given by:

(15a) $$\left[\begin{array}{cc} A_{11} & A_{12}\\ -A_{12} & A_{22} \end{array}\right]\left[\begin{array}{c} 1\\ \gamma \end{array}\right]=0$$

(15b) $$A_{11}=\left(1+j\omega \beta \right)\left(D\lambda^{4}-\frac{2B}{r}\lambda^{2}+\frac{A}{r^{2}}\right)-\omega^{2}m+j\omega m\alpha$$

(15c) $$A_{12}=\left(1+j\omega \beta \right)\left(-\left(B+\frac{D}{r}\right)\lambda^{3}+\frac{1}{r}\left(A+\frac{B}{r}\right)\lambda \right)$$

(15d) $$A_{22}=-\left(1+j\omega \beta \right)\left(A+\frac{2B}{r}+\frac{D}{r^{2}}\right)\lambda^{2}-\omega^{2}m+j\omega m\alpha$$

The conditions for the existence of the nontrivial solution of Equation (15) requires that the determinant of the coefficient matrix of Equation (15) must be zero which yields a characteristic equation for wavenumber λ.

(16) $$C_{3}\lambda^{6}+C_{2}\lambda^{4}+C_{1}\lambda^{2}+C_{0}=0$$

Solving Equation (16) for λ, the general solution of Equation (14) is shown as follows.

(17a) $$\tilde{w}=\varphi a,$$

(17b) $$\tilde{u}=\left(\gamma \cdot \varphi \right)a$$

(17c) $$a={\left[\begin{array}{cccccc} a_{1} & a_{2} & a_{3} & a_{4} & a_{5} & a_{6} \end{array}\right]}^{T}$$

(17d) $$\lambda =\left[\begin{array}{cccccc} \lambda_{1} & \lambda_{2} & \lambda_{3} & \lambda_{4} & \lambda_{5} & \lambda_{6} \end{array}\right]$$

(17e) $$\gamma =\left[\begin{array}{cccccc} \gamma_{1} & \gamma_{2} & \gamma_{3} & \gamma_{4} & \gamma_{5} & \gamma_{6} \end{array}\right],$$

(17f) $$\gamma_{k}=\frac{A_{12}}{A_{22}}{│}_{\lambda =\lambda_{k}}$$

(17g) φ=exp(λs)

where (⋅) represents element-wise product operator.

For a segment no. *i*, the solution must satisfy displacement and force/moment boundary conditions at both endpoints (s=0 and $s=S_{i}$, Figure 2). Defining $\hat{d}\left(s\right)={\left[\begin{array}{ccc} \tilde{w} & \tilde{u} & \tilde{\psi } \end{array}\right]}^{T}$ one can get, from Equation (17) and considering that $\tilde{\psi }=\partial \tilde{w}/\partial s-\tilde{u}/r$, the following expression.

(18) $$\hat{d}\left(s\right)=\left[\begin{array}{c} \tilde{w}\\ \tilde{u}\\ \tilde{\psi } \end{array}\right]=\left[\begin{array}{c} \varphi \\ \gamma \cdot \varphi \\ \lambda \cdot \varphi -\frac{1}{r}\gamma \cdot \varphi \end{array}\right]a$$

Defining $\hat{f}\left(s\right)={\left[\begin{array}{ccc} \tilde{V} & \tilde{N} & -\tilde{M} \end{array}\right]}^{T}$ where $\tilde{N}$ and $\tilde{M}$ are the complex amplitudes of N and M (given by Equations (A1)) and $\tilde{V}=\partial \tilde{M}/\partial s$ is the complex amplitude of the shear force V, one can substitute (17a) into (A1) to get

(19) $$\hat{f}\left(s\right)=\left[\begin{array}{c} \tilde{V}\\ \tilde{N}\\ -\tilde{M} \end{array}\right]=\left(1+j\omega \beta \right)\left[\begin{array}{c} \frac{B}{r}\lambda \cdot \varphi -D\lambda .^{3}\cdot \varphi +\left(B+\frac{D}{r}\right)\lambda .^{2}\cdot \gamma \cdot \varphi \\ \left(\frac{A}{r}\varphi -B\lambda .^{2}\cdot \varphi \right)+\left(A+\frac{B}{r}\right)\lambda \cdot \gamma \cdot \varphi \\ -\left(\frac{B}{r}\varphi -D\lambda .^{2}\cdot \varphi \right)-\left(B+\frac{D}{r}\right)\lambda \cdot \gamma \cdot \varphi \end{array}\right]a+\left[\begin{array}{c} 0\\ \vartheta_{N}{\tilde{v}}^{\left(i\right)}\\ -\vartheta_{M}{\tilde{v}}^{\left(i\right)} \end{array}\right]$$

In Equation (19): $\lambda^{n}=\left[\begin{array}{cccccc} \lambda_{1}^{n} & \lambda_{2}^{n} & \lambda_{3}^{n} & \lambda_{4}^{n} & \lambda_{5}^{n} & \lambda_{6}^{n} \end{array}\right]$

${\tilde{v}}^{\left(i\right)}$ is the complex amplitude of the voltage generated by the segment (no. *i*), derived by placing Equation (13) into Equation (12) and re-arranging:

(20) $${\tilde{v}}^{\left(i\right)}=\frac{be_{31}}{C_{p}+1/j\omega Z\left(\omega \right)}\left({\tilde{u}]}_{0}^{S_{i}}+\int_{0}^{S_{i}}\frac{\tilde{w}}{r}\;ds-z_{pc}{\frac{\partial \tilde{w}}{\partial s}]}_{0}^{S_{i}}\right)$$

Substituting Equation (17) into Equation (20)

(21) $${\tilde{v}}^{\left(i\right)}=\frac{be_{31}}{C_{p}+1/j\omega Z\left(\omega \right)}\left({\gamma \cdot \varphi ]}_{0}^{S_{i}}+\frac{1}{r}{\lambda .^{-1}\cdot \varphi ]}_{0}^{S_{i}}-z_{pc}{\lambda \cdot \varphi ]}_{0}^{S_{i}}\right)a=C^{\left(i\right)}a$$

It should be noted that in the specific cases considered in this paper, the electrical load is a pure resistor $R_{L}$, i.e., $Z\left(\omega \right)=R_{L}$. However, the use of the generic impedance term Z allows application to more complicated external circuits comprising resistors, capacitors, and inductances. Such complex circuits are useful in energy harvesting to maximize the power generated through electrical impedance matching [41,42], or even to achieve simultaneous energy harvesting and ambient vibration attenuation through a tuned shunted circuit [18].

With reference to Figure 2, the nodal degree-of-freedom vector $d^{\left(i\right)}$ and corresponding vector of forces and moments $f^{\left(i\right)}$ for segment no. *i* are defined as

(22) $$d^{\left(i\right)}={\left[\begin{array}{cccccc} {\tilde{w}}_{i} & {\tilde{u}}_{i} & {\tilde{\psi }}_{i} & {\tilde{w}}_{i+1} & {\tilde{u}}_{i+1} & {\tilde{\psi }}_{i+1} \end{array}\right]}^{T}=\left[\begin{array}{c} \hat{d}\left(0\right)\\ \hat{d}\left(S_{i}\right) \end{array}\right]=A^{\left(i\right)}a$$

(23) $$f^{\left(i\right)}={\left[\begin{array}{cccccc} {\tilde{F}}_{w_{i}} & {\tilde{F}}_{u_{i}} & {\tilde{M}}_{\psi_{i}} & {\tilde{F}}_{w_{i+1}} & {\tilde{F}}_{u_{i+1}} & {\tilde{M}}_{\psi_{i+1}} \end{array}\right]}^{T}=\left[\begin{array}{c} -\hat{f}\left(0\right)\\ +\hat{f}\left(S_{i}\right) \end{array}\right]=B^{\left(i\right)}a$$

Finding a from Equation (22) and substituting it into Equation (23)

(24) $$f^{\left(i\right)}=B^{\left(i\right)}{\left(A^{\left(i\right)}\right)}^{-1}d^{\left(i\right)}=D^{\left(i\right)}d^{\left(i\right)}$$

where $D^{\left(i\right)}$ is the dynamic stiffness matrix of segment no. *i*.

If lumped inertias of masses $M_{i}$ and $M_{i+1}$ and moments of inertia $I_{i}$ and $I_{i+1}$ are attached to end nodes nos. i and i+1 of segment no. i, the following matrix is added to its dynamic stiffness matrix $D^{\left(i\right)}$ [26]:

(25a) $$D_{\mathrm{inertia}}^{\left(i\right)}=\left[\begin{array}{cc} D_{\mathrm{inertia},\;\mathrm{node}\;\mathrm{no}.\;i}^{\left(i\right)} & 0\\ 0 & D_{\mathrm{inertia},\;\mathrm{node}\;\mathrm{no}.\;(i+1)}^{\left(i\right)} \end{array}\right]$$

(25b) $$D_{\mathrm{inertia},\;\mathrm{node}\;\mathrm{no}.\;i}^{\left(i\right)}=\left[\begin{array}{ccc} \left(-\omega^{2}+j\omega \alpha \right)M_{i} & 0 & 0\\ 0 & \left(-\omega^{2}+j\omega \alpha \right)M_{i} & 0\\ 0 & 0 & -\omega^{2}I_{i} \end{array}\right]$$

To avoid duplication of inertia, a given attached lumped inertia should be either added only to one segment, or shared (in any arbitrary proportion) between contiguous segments.

Now considering $T^{\left(i\right)}$ to be transformation matrix between global and local coordinates (Figure 2),

(26a) $${\bar{d}}^{\left(i\right)}=T^{\left(i\right)}d^{\left(i\right)},$$

(26b) $${\bar{f}}^{\left(i\right)}=T^{\left(i\right)}f^{\left(i\right)}$$

(26c) $$T^{\left(i\right)}=\left[\begin{array}{cc} R\left(\theta_{i}\right) & 0\\ 0 & R\left(\theta_{i+1}\right) \end{array}\right],$$

(26d) $$R\left(\theta \right)=\left[\begin{array}{ccc} \cos\left(\theta \right) & \sin\left(\theta \right) & 0\\ \sin\left(\theta \right) & -\cos\left(\theta \right) & 0\\ 0 & 0 & 1 \end{array}\right]$$

where $\left(\theta_{i}\right)$ is the angle between tangent at node no. i and global x axis.

The global dynamic stiffness matrix of the segment is therefore

(27) $${\bar{D}}^{\left(i\right)}=T^{\left(i\right)}D^{\left(i\right)}{\left(T^{\left(i\right)}\right)}^{-1}$$

### 2.3. Matrix Assembly and Frequency Response Functions

Due to symmetry of the energy harvester configuration in Figure 1a, the modelling need only consider one of the transducers, each carrying half the tip mass and shunted across twice the external circuit resistance $R_{\mathrm{ext}}$ (i.e., in previous equations, $Z\left(\omega \right)=R_{L}=2R_{\mathrm{ext}}$ since transducers are connected in parallel across $R_{\mathrm{ext}}$). With reference to Figure 1b, a total of seven segments were used to model the device. Five segments—2, 3, 4, 5, and 6—were used to model the region between the base joint and the tip joint (both joints coinciding with the centers of the radial bearings which act as pivots). Segments 1 and 2, and 6 and 7, correspond to the clamped regions and were therefore considered rigid and their density was chosen such that their moment of inertia (MoI) about their respective joint axis matches with that of the corresponding clamp, alternatively this clamp inertia could be applied using Equation (25) but for the sake of using a unified model for both DSM and ANSYS it is avoided; any slight excess clamp mass arising from choosing the density on the basis of its MoI is corrected by correspondingly adjusting the value of the tip mass (Figure 1) which is included using Equation (25) with $I_{i}$ set to zero. Segments 3 and 5 correspond to regions of steel substrate extent that are not covered by the clamp—this gap region is necessary to avoid risk of cracking the piezoelectric material and is considered with edge length of 3 mm. Notice that the mid-surfaces of these short steel segments are not continuous with the mid-surface of the contiguous composite segment no.4. This introduces a slight error in the model since the matrix assembly process described below assumes continuity of the mid-surface. However, the effect of this error is not considered significant (as indeed evidenced by verification against ANSYS in later sections) since the beam is thin. Using a matrix assembly process based on continuity of displacement at adjacent ends of contiguous segments, the dynamic stiffness matrix $\bar{D}$ of the whole system with 7 segments (Figure 1b) is obtained:

(28a) $$\bar{f}=\bar{D}\bar{d}$$

(28b) $$\bar{d}=\left[\begin{array}{c} {\bar{d}}^{\left(1\right)}\\ \vdots \\ {\bar{d}}^{\left(8\right)} \end{array}\right],\;$$

(28c) $$\bar{f}=\left[\begin{array}{c} {\bar{f}}^{\left(1\right)}\\ \vdots \\ {\bar{f}}^{\left(8\right)} \end{array}\right]$$

(28d) $${\bar{f}}^{\left(i\right)}={\left[\begin{array}{cccccc} {\tilde{F}}_{x_{i}} & {\tilde{F}}_{y_{i}} & {\tilde{M}}_{\psi_{i}} & {\tilde{F}}_{x_{i+1}} & {\tilde{F}}_{y_{i+1}} & {\tilde{M}}_{\psi_{i+1}} \end{array}\right]}^{T},$$

(28e) $$\;{\bar{d}}^{\left(i\right)}={\left[\begin{array}{ccccccc} {\tilde{x}}_{i} & {\tilde{y}}_{i} & {\tilde{\psi }}_{i} & \ldots & {\tilde{x}}_{i+1} & {\tilde{y}}_{i+1} & {\tilde{\psi }}_{i+1} \end{array}\right]}^{T}$$

In Equation (28), $\bar{d}$ and $\bar{f}$ contain the complex amplitudes of the global nodal displacements at node nos. *i* ($x_{i}$, $y_{i}$, $\psi_{i}$) and the corresponding excitations ($F_{x_{i}}$, $F_{y_{i}}$, $M_{\psi_{i}}$), i=1…8. The tip mass matrix (Equation (25)) is added to the rows and columns corresponding to node no. 7 (Figure 1b).

The boundary conditions are as follows:

(29a) y=0 @ node no. 2

(29b) y=0 @ node no.7

The receptance matrix α then relates $\bar{d}$ to $\bar{f}$ as follows

(30) $$\bar{d}=\alpha \bar{f}$$

where α is determined in the following way:

- the rows relating to ${\tilde{y}}_{2}$ and ${\tilde{y}}_{7}$, and columns relating to ${\tilde{F}}_{y_{2}}$ and ${\tilde{F}}_{y_{7}}$ are all padded with zeros due to the boundary conditions;
- the remainder of α is $\alpha_{\mathrm{red}}={\bar{D}}_{\mathrm{red}}^{-1}$ where ${\bar{D}}_{\mathrm{red}}$ is obtained from $\bar{D}$ by eliminating the columns relating to ${\tilde{y}}_{2}$ and ${\tilde{y}}_{7}$, and the rows relating to ${\tilde{F}}_{y_{2}}$ and ${\tilde{F}}_{y_{7}}$.

Now, with reference to Figure 1, the only effective non-zero excitation in $\bar{f}$ is ${\tilde{F}}_{x_{2}}$, since ${\tilde{F}}_{y_{2}}$ are ${\tilde{F}}_{y_{7}}$ are non-effective due to the boundary conditions (notice also that ${\tilde{F}}_{x_{7}}=0$ since the inertia and damping effect from the tip mass at node no. 7 are considered in Equation (25) and the equivalent damping fit of Equation (8)).

Hence, the transmissibility of the device, relating the harmonic displacements/velocities/accelerations at tip (node no. 7) to the base (node no. 2) is given by:

(31) $$T=\frac{{\tilde{x}}_{7}}{{\tilde{x}}_{2}}=\frac{\alpha_{{\tilde{x}}_{7},\;{\tilde{F}}_{x_{2}}\;}}{\alpha_{{\tilde{x}}_{2},\;{\tilde{F}}_{x_{2}}\;}}$$

where $\alpha_{{\tilde{x}}_{7},\;{\tilde{F}}_{x_{2}}\;}$ and $\alpha_{{\tilde{x}}_{2},\;{\tilde{F}}_{x_{2}}\;}$ are the receptance matrix terms relating the displacements at nodes 7 and 2, respectively, with the transmitted force at the base.

The voltage is produced by segment no. 4 (Figure 1b). Finding a from Equation (22) and substituting it in Equation (21) gives

(32) $${\tilde{v}}^{\left(4\right)}=C^{\left(4\right)}{\left(A^{\left(4\right)}\right)}^{-1}d^{\left(4\right)}=G^{\left(4\right)}d^{\left(4\right)}=G^{\left(4\right)}{\left(T^{\left(4\right)}\right)}^{-1}{\bar{d}}^{\left(4\right)}$$

From Equation (20):

(33) $${\bar{d}}^{\left(4\right)}={\tilde{F}}_{x_{2}}\alpha_{{\bar{d}}^{\left(4\right)},{\tilde{F}}_{x_{2}}}$$

where $\alpha_{{\bar{d}}^{\left(4\right)},\;{\tilde{F}}_{x_{2}}}$ is that sub-column of α relating the complex amplitudes of nodal degrees of freedom of segment no. 4, contained in ${\bar{d}}^{\left(4\right)}$, with the complex amplitude of the transmitted force at the base joint ${\tilde{F}}_{x_{2}}\;{\tilde{F}}_{x_{2}}$. Substituting Equation (33) into Equation (32), the frequency response function (FRF) relating ${\tilde{v}}^{\left(4\right)}$ to ${\tilde{F}}_{x_{2}}$ is written as

(34) $$\frac{{\tilde{v}}^{\left(4\right)}}{{\tilde{F}}_{x_{2}}}=G^{\left(4\right)}{\left(T^{\left(4\right)}\right)}^{-1}\alpha_{{\bar{d}}^{\left(4\right)},\;{\tilde{F}}_{x_{2}}}$$

The voltage FRF relating ${\tilde{v}}^{\left(4\right)}$ to the base acceleration (normalised by $g=9.81m/s^{2}$) is then written as

(35) $$\frac{{\tilde{v}}^{\left(4\right)}}{\left(-\omega^{2}{\tilde{x}}_{2}\right)/g}=\frac{gG^{\left(4\right)}{\left(T^{\left(4\right)}\right)}^{-1}\alpha_{{\bar{d}}^{\left(4\right)},\;{\tilde{F}}_{x_{2}}}}{-\omega^{2}\alpha_{{\tilde{x}}_{2},\;{\tilde{F}}_{x_{2}}}}$$

The power FRF is calculated from voltage FRF using relations

(36) $$\;\;\mathrm{power}\;\mathrm{FRF}=\frac{{\left|\mathrm{voltage}\;\mathrm{FRF}\right|}^{2}}{R_{L}}$$

## 3. Finite Element Modelling Using Ansys

**Figure 3:**
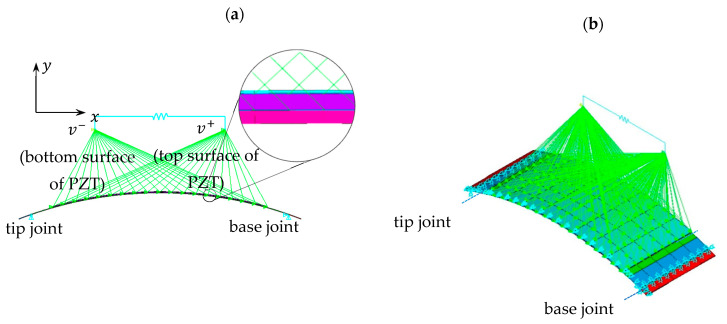
Meshed model (coarse): (**a**) plane (**b**) solid.

Finite element (FE) modelling was performed using ANSYS (ANSYS Academic Research Mechanical, Release 19.1). The ANSYS elements that support the piezoelectric effect are PLANE223 (Figure 3a) and SOLID226 (Figure 3b). These were used in the piezoelectric layer. For the non-piezoelectric layers, they were replaced by their non-piezoelectric counterparts (PLANE183 and SOLID186, respectively). Both piezoelectric element types are compatible with CIRCU94 element, which is used to model external circuit (resistors in this case). In order to model electrodes of PZT patch, defined nodes on the upper surface and bottom surface of the piezoelectric layer are coupled into two separate sets. The PLANE223/183 elements lie in the *x*-*y* plane (Figure 3a) and have unit depth in the *z*-direction. Therefore, the value of resistance should be multiplied by *b* (the width of the THUNDER), and the tip mass should be divided by *b*, while modelling with this (plane-type) element. Furthermore, for the model with PLANE223/183 elements, the Poisson ratio effect applies only in the *x*-*y* plane (the plane of the elements), i.e., does not apply along the *z*-direction. Hence, the model with PLANE223/183 elements shown in Figure 3a will be equivalent to a model based on beam elements in terms of deformation behavior (i.e., curvature in *x*-*y* plane only).

**Figure 4:**
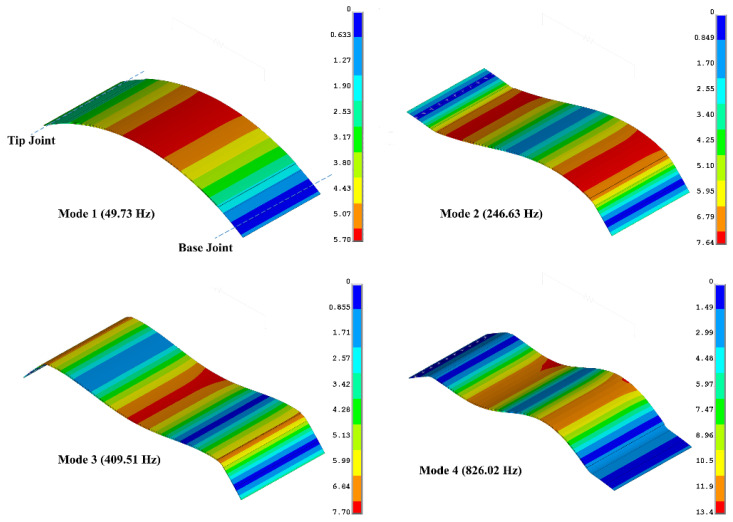
Mode shapes of THUNDER transducer section of device in Figure 1, using solid elements.

For the purpose of investigating mesh convergence, a purely mechanical setup (i.e., with the external resistance short-circuited) was considered, and a modal analysis was conducted to obtain and compare undamped natural frequencies of the device in Figure 1 using different element types. In addition to aforementioned PLANE223/183 and SOLID226/186, the element type SHELL281 (which does not support the piezoelectric effect) was also considered for the purely mechanical exercise (there is no piezoelectric shell-type element in ANSYS). All of these elements use quadratic shape functions to interpolate field variables. MASS21 was used to model the tip mass. For the 3D models (solid or shell elements), a symmetry condition was applied in the *z*-plane. For the free vibration analysis, the boundary conditions were applied as shown in Figure 3, i.e., zero displacement in *z*-direction at all nodes, zero displacement in *y* direction at tip and base joints, zero displacement in *x*-direction at base joint. The values for the first four natural frequencies of the device using the different types of ANSYS elements are presented in Table 3 (columns 2–6). The corresponding modal displacement shapes of the transducer section of the device modelled using solid elements are depicted in Figure 4. Convergence was achieved for an element edge length of 2 mm in circumferential direction, and for plane and solid models one element was enough through thickness of each layer. For shell and solid models, 3 mm of element edge length was enough in *z* direction. The number of elements used in each model for plane, shell, and solid elements were 350, 1211, and 4565, respectively. Figure 4 shows that the transducer section of the device vibrates approximately like a beam in the first four modes, with curvature being mainly in the *x*-*y* plane (particularly the first three modes). With reference to Table 3, the plane element results (which effectively describe beam-bending behavior of the THUNDER in the *x*-*y* plane, as explained above) are consistent with both the shell and plane results since the ~3 to ~5% shortfall in the natural frequencies can be attributed to the effective bending stiffness of each layer of the shell and solid element models being $1/\left(1-\nu^{2}\right)$ times that of a beam-bending model, where ν is the Poisson ratio of the layer [43]. In fact, neglecting the adhesive layer, assuming an average Poisson ratio of 0.3, and considering that the natural frequencies are approximately proportional to the square root of the bending stiffness, the percentage difference in natural frequencies between the plane element model and the shell or solid element models is expected to be $100\left(\sqrt{1-\nu^{2}}-1\right)=-4.6$, which agrees with the percentage differences for plane vs. shell and plane vs. solid in Table 3.

The last two columns of Table 3 respectively show the DSM results for the undamped natural frequencies and the percentage differences relative to the ANSYS plane element results. It is clear that the ANSYS plane element results are much closer to the DSM results than to the ANSYS shell or solid element results. This is expected since the DSM model is directly based on beam-bending, and the ANSYS plane element model describes a similar beam-bending behavior, albeit modelled indirectly through plane elements (resulting in the small differences in the last column of Table 3). It should also be noted that, as seen from Section 2, DSM is a frequency domain method used to derive FRFs. Hence, unlike FE (ANSYS), the undamped natural frequencies in DSM are not found by solving an eigenvalue problem since the final relation is a system of equations (Equation (30)) relating the nodal displacements and forces in the frequency domain (rather than an equation of motion in the time domain involving discrete mass and stiffness matrices). However, the undamped natural frequencies can be determined by DSM from the frequency locations of the peaks of the transmissibility FRF (Equation (31)) under zero damping conditions. For zero electrical load and no material damping, these resonance peaks would be infinite, and their frequencies are more accurately located by noting the zero crossing points of the denominator of Equation (31) (i.e., the FRF $\alpha_{{\tilde{x}}_{2},\;{\tilde{F}}_{x_{2}}\;}\left(\omega \right)$). It should also be noted that, although the DSM undamped natural frequencies are determined from the FRF, the DSM is still regarded as an analytical method since the FRF terms are based on the analytical solution (Equation (17)) of the dynamic equations of the curved beam segment.

## 4. Experimental Testing 

In this section, the experimental setup is presented (Section 4.1) followed by a description of the estimation of the damping in the energy harvesting device (Section 4.2).

### 4.1. Experimental Setup

**Figure 5:**
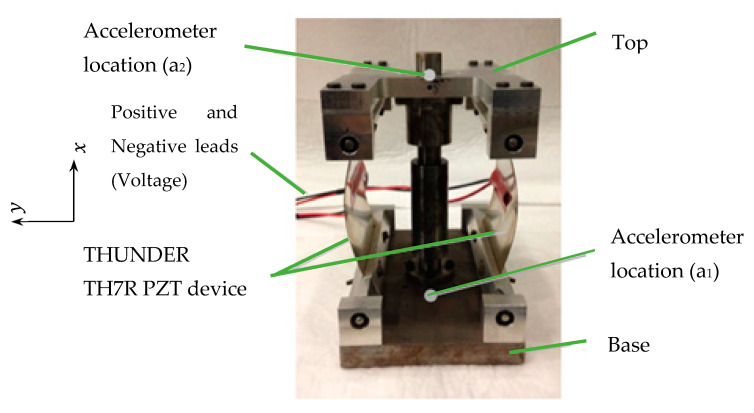
Photograph of the vibration energy harvesting device.

**Figure 6:**
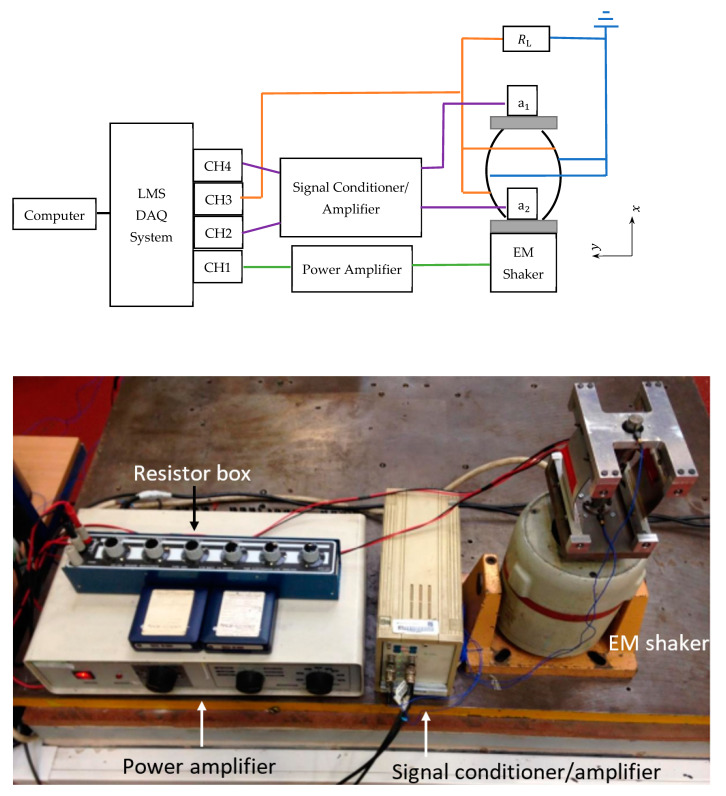
Schematic diagram of the experimental setup and its instrumentation.

Figure 5 shows an annotated photograph of the energy harvesting device and Figure 6. shows the experimental setup. The experimental setup was comprised of the following equipment.

- A PC-controlled data acquisition (DAQ) system (not shown in Figure 6) consisting of hardware (LMS Scadas 5) and spectral analysis software (LMS Test.Lab).
- Two accelerometers (PCB 352C22), one attached to the base of the energy harvesting device and the other attached to its tip mass.
- A signal conditioner/amplifier, to remove the noise from signals received from accelerometers and amplify them before channeling to the DAQ system.
- An electromagnetic (EM) shaker on which the energy harvesting device was mounted.
- A power amplifier to control the gain of the random excitation signal output from the DAQ system, which was then fed to the EM shaker.
- A resistor box which was controlled manually to apply purely resistive electrical loads in the range 0.5–500 kΩ.

The signal delivered to the shaker was bandlimited white noise with frequency bandwidth set to 0–300 Hz. In order to obtain transmissibility and voltage FRFs (frequency response functions), the accelerations at the base and top of the energy harvesting device in Figure 5, and piezoelectric voltage need to be measured. The base accelerometer ($a_{1}$) is connected to channel no. 2 (CH2) of the DAQ system and is set up as the reference signal (its output provides the denominator of the transmissibility and voltage FRFs). The top accelerometer ($a_{2}$) is connected to CH4. A resistance box is connected across the piezoelectric elements and the voltage difference across the resistance is fed to CH3. The required FRFs were generated by the DAQ system spectral analysis software from the acceleration and voltage responses channeled to the DAQ system. 

### 4.2. Estimation of Damping

To estimate the damping ratio from the experimental data, we approximate the device as a base-excited single-degree-of-freedom system, for which the transmissibility can be expressed as a function of excitation frequency ω, the undamped natural frequency $\omega_{n}$, and viscous damping ratio ζ:

(37) $$T=\frac{1+2\frac{\omega }{\omega_{n}}\zeta j}{1-\frac{\omega^{2}}{\omega_{n}^{2}}+2\frac{\omega }{\omega_{n}}\zeta j}$$

**Figure 7:**
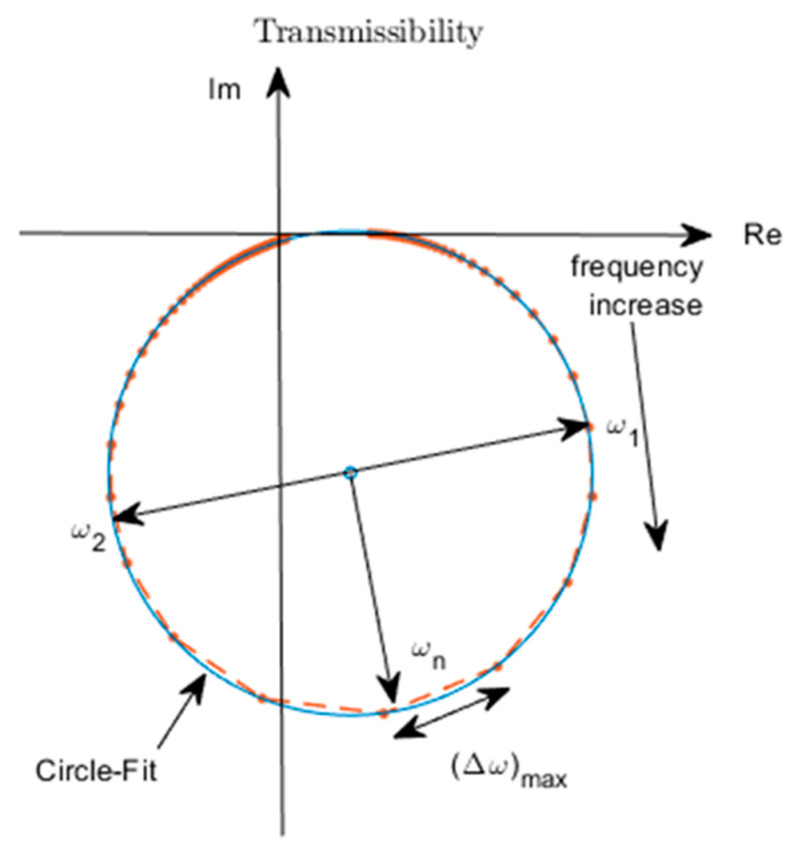
Nyquist plot of transmissibility function for single degree of freedom system (Equation (37)).

From Equation (37), it is seen that the expression for $T-1={\left(\omega /\omega_{n}\right)}^{2}/\left[1-{\left(\omega /\omega_{n}\right)}^{2}+2\left(\omega /\omega_{n}\right)\zeta j\right]$ is of the same form as the accelerance (inertance) FRF of a viscously damped single-degree-of-freedom system [44]. Hence, the graph of Im{T−1} (*y*-axis) vs. Re{T−1} (*x*-axis) (i.e., the Nyquist plot of T−1) will be approximately circular, just like for the accelerance FRF [45]. The Nyquist plot of T will be the same as that for T−1 but shifted by 1 unit to the right along the horizontal axis, as shown in Figure 7.

Three approaches based on the expression in Equation (37) were used to estimate the damping ratio from the experimental transmissibility data. 

The first approach considered that

(38a) Re{T(ω=ωn)}=1

(38b) Im{T(ω=ωn)}=−12ζ 

Equation (38a) was used to locate the resonance point ($\omega =\omega_{n}$), thus enabling ζ to be determined from Equation (38b).

The second approach considered that, $\left|T\left(\omega =\omega_{n}\right)-1\right|=1/\left(2\zeta \right)$, the quality factor [45]. Hence, the half-power points are defined as two frequency points $\omega_{1}$ and $\omega_{2}$ (assuming $\omega_{2}>\omega_{1}$) on either side of $\omega_{n}$, for which

(39) $${\left|T\left(\omega_{1}\right)-1\right|}^{2}={\left|T\left(\omega_{2}\right)-1\right|}^{2}=\frac{1}{2}{\left|T\left(\omega =\omega_{n}\right)-1\right|}^{2}$$

Substituting Equation (37) into Equation (39), these frequencies are determined from the following equation:

(40a) $${\left(\frac{\omega }{\omega_{n}}\right)}^{4}-2{\left(\frac{\omega }{\omega_{n}}\right)}^{2}\left(1-2\zeta^{2}\right)+1-8\zeta^{2}=0,$$

(40b) $$\omega =\omega_{1},\omega_{2}$$

Considering small damping $\left(\zeta <\frac{1}{2\sqrt{2}}\right)$ and neglecting terms in $\zeta^{2}$ and higher order terms, the standard half-power point relation is obtained:

(41) $$\frac{\omega_{2}-\omega_{1}}{\omega_{n}}=2\zeta$$

Hence, after determining the two frequencies for which Equation (40) applies, ζ is determined from Equation (41).

The third approach for estimating the damping was to perform a circle-fit of the Nyquist plot of the experimental transmissibility data. Similar to the Nyquist plots of the receptance/mobility accelerance FRFs of viscously damped single-degree-of-freedom systems [44], the resonance point $\omega =\omega_{n}$ was identified by the location where the angular spacing of the data points was a maximum and the half power points $\omega =\omega_{1},\;\omega_{2}$ subsequently determined by displacing 90° on either side of the resonance frequency location, as shown in Figure 7.

The damping values obtained using these three methods are presented in Table 4 for different magnitudes of the external electrical load. The damping ratio estimates by the three methods are in good agreement, however the circle-fit method is expected to be the most accurate since it is less prone to errors introduced by the frequency resolution of the data and measurement noise.

With reference to the results in Table 4, for any given method, the damping ratios are minimal at the lowest resistance (approximate short circuit) and highest resistance (approximate open circuit). In these cases, the damping ratio ζ is due to the mechanical effects only since very little electrical power is dissipated due to the resistance being very low (short circuit) or the current being very low (open circuit). In between these two extremes, the estimated damping ratio is higher since it contains the additional damping effect introduced by the energy harvesting. It should also be noted that the undamped natural frequency $\omega_{n}$ in Equation (37) will vary with the magnitude of the electrical load (since the electrical load affects the effective stiffness, apart from the damping). The transmissibility resonance condition was defined above as $\omega =\omega_{n}$. For 0<ζ<0.1 (as in the short and open circuit conditions, Table 4) this frequency is very close (but not identical to) the frequency at which the transmissibility is maximum. However, given that the energy harvesting effect raises ζ to a value close to 0.1, it is important to make the distinction between the two frequencies. Hence, for the remainder of the paper the following terms are used to distinguish from the condition $\omega =\omega_{n}$:

- Transmissibility resonance peak frequency—frequency at which transmissibility FRF magnitude is maximum;
- Voltage FRF resonance peak frequency—frequency at which the voltage FRF magnitude is maximum.

It is also noted that, in the analysis results presented in the following section, the value used for β ($=2\zeta_{1}/\omega_{1}$, Equation (8)) uses values of $\zeta_{1}$ and $\omega_{1}$ obtained from experimental data for the short circuit condition (lowest resistance in Table 4). This value is used in the DSM, as well as in the FE as the stiffness-proportional damping multiplier ([C]=β[K]).

## 5. Presentation of FRF Results and Discussion

### 5.1. DSM vs. 2D/3D ANSYS vs. Experiment

**Figure 8:**
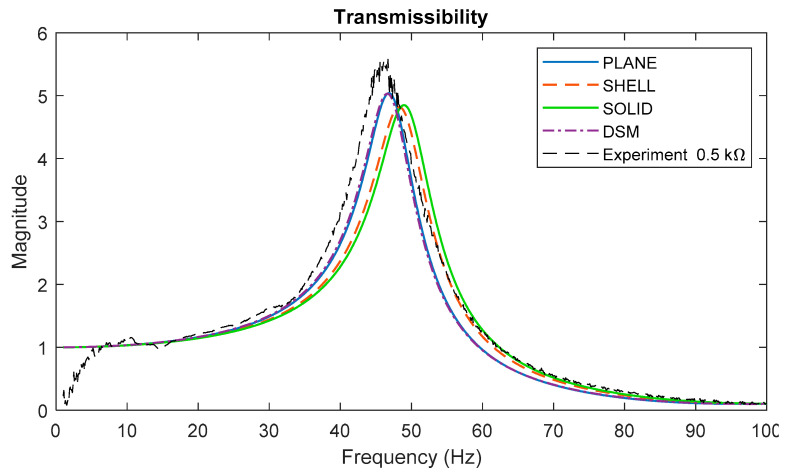
Predicted transmissibility using various models for the case of short-circuited load (no electrical effect), together with the experimental transmissibility obtained at the lowest resistance considered (close to short circuit).

A harmonic response analysis was performed in ANSYS to obtain the transmissibility FRF using the different types of elements. With reference to Figure 1 and Figure 3, the boundary condition for the *x*-displacement amplitude of the base was set to unity and the *x*-displacement amplitude of the tip was therefore considered as the transmissibility. Figure 8 shows the predicted transmissibility FRFs by the different FE models, together with DSM prediction given by Equation (31). These predictions are for the case of zero electrical load (pure short circuit). The experimental result for the lowest electrical load considered (0.5 kΩ) is also included; although the experimental load is not zero, it is low enough to be considered as “short circuit” in practice. The resonance frequencies of the plane, shell, and solid element model transmissibility results in Figure 8 are very close to those already given in Table 3 since the system is relying only on the mechanical damping and the resonance frequency is weakly affected by damping if the damping ratio is less than 0.1. The result from the DSM model agrees very closely with that from the plane element model, as expected since both models describe a beam-bending behavior.

**Figure 9:**
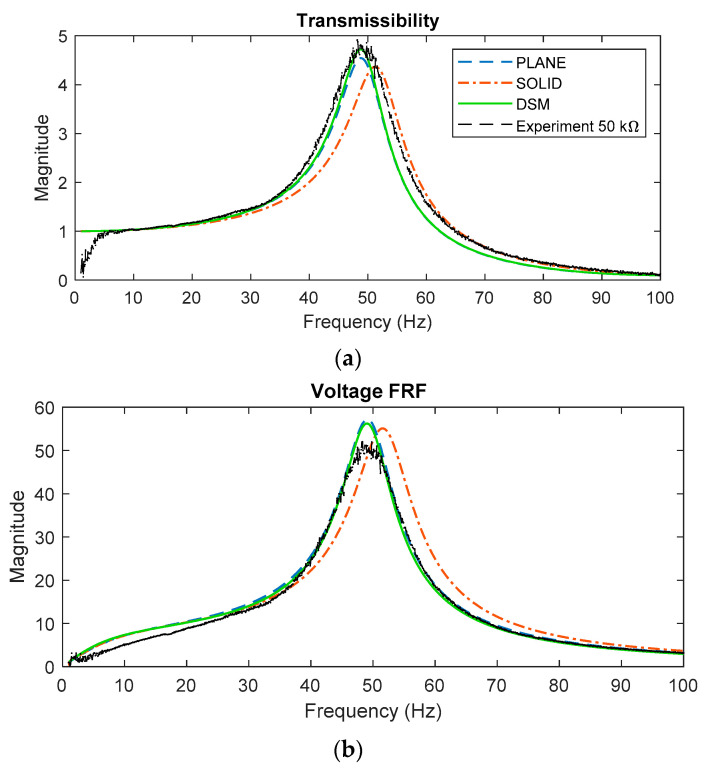
Predicted transmissibility (**a**) and voltage FRF (**b**) using three different models for the case of 50 kΩ resistance, together with the corresponding experimental results.

Next, the piezoelectric effect is introduced into the models by applying a significant magnitude of electrical resistance. Figure 9 shows the predicted transmissibility and voltage FRF using three different models for the case of 50 kΩ resistance, together with the corresponding experimental results. The peaks of the transmissibility FRFs in Figure 9 are all seen to be lower than those in Figure 8, due to the additional damping introduced by the piezoelectric effect. There is again very close agreement between the DSM and plane element model predictions, both with regard to transmissibility and voltage FRF (the DSM voltage FRF being computed using Equation (35)). 

Considering Figure 8 and Figure 9, all models agree reasonably well with the experimental results, but the beam-bending ones (DSM, FE plane element) provide better agreement (than the FE solid element model) since their resonance frequencies are closer to the experimental one. The likely reason why the beam-bending model resonance frequency is ~5% lower than that from the solid element model was already discussed above. The fact that the beam-bending model resonance is closer to the experimental resonance (than the FE solid element model) is somewhat unexpected but can be attributed to random uncertainties in the various modelling parameters. It should also be noted that the experiments were conducted with the device (Figure 1) vertically oriented, as shown in Figure 6, to minimize sliding friction in the linear bearing; this means that the static load from the weight of the tip mass (~4 N) slightly increased the curvature of the transducers—the increased curvature would have the effect of slightly lowering the fundamental resonance frequency of the device [38].

### 5.2. DSM and 2D ANSYS vs. Experiment over a Range of Electrical Loads 

**Figure 10:**
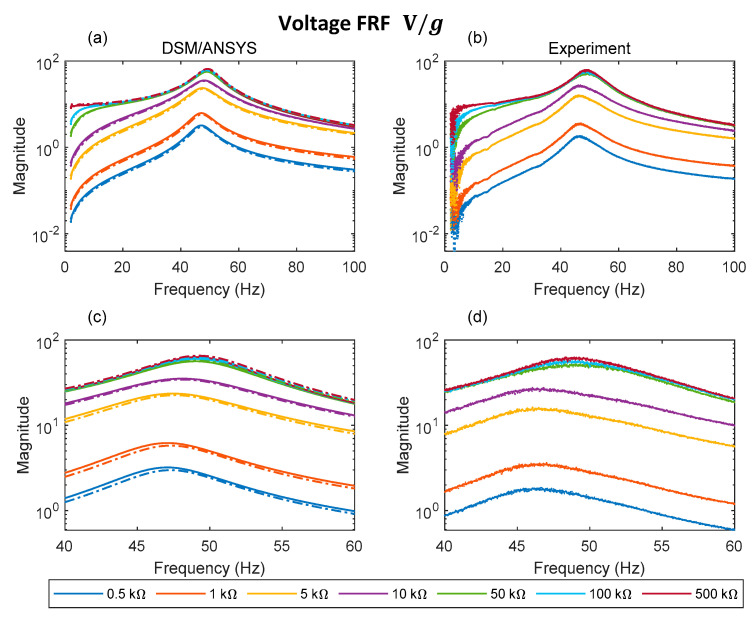
Analytical and experimental voltage FRF for different electrical loads: (**a**) DSM/ANSYS; (**b**) experiment; (**c**) DSM/ANSYS result zoomed in resonance region; (**d**) experimental result zoomed in resonance region (dashed and solid lines in (**a**,**c**) represent data associated with ANSYS plane element model and DSM, respectively).

**Figure 11:**
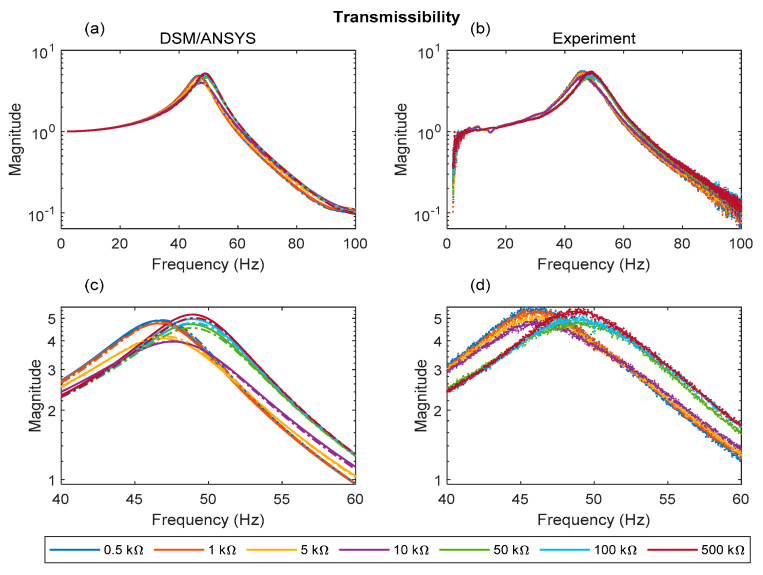
Analytical and experimental transmissibility FRF for different electrical loads: (**a**) DSM/ANSYS; (**b**) experiment; (**c**) DSM/ANSYS result zoomed in resonance region; (**d**) experimental result zoomed in resonance region (dashed and solid lines in (**a**,**c**) represent data associated with ANSYS plane element model and DSM, respectively).

**Figure 12:**
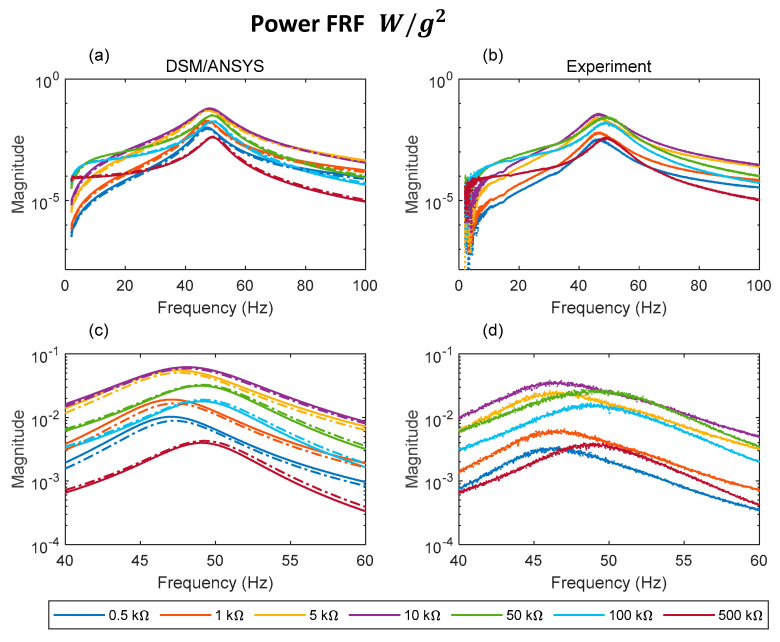
Analytical and experimental power FRFs for different loads: (**a**) DSM/ANSYS; (**b**) experiment; (**c**) DSM/ANSYS result zoomed in resonance region; (**d**) experimental result zoomed in resonance region (dashed and solid lines in (**a**,**c**) represent data associated with ANSYS plane element model and DSM, respectively).

Figure 10a–d, Figure 11a–d and Figure 12a–d, respectively, show the voltage FRF magnitude, transmissibility magnitude, and the power FRF over a range of electrical loads. With reference to the predictions (Figure 10a,c, Figure 11a,c and Figure 12a,c) it is evident that both theoretical results (DSM, ANSYS) show very close agreement. The ANSYS results presented are those from the plane element model, since these give the best agreement with DSM and experiment as shown in Section 5.1.

Focusing on the voltage FRFs (Figure 10a–d) it can be observed that by increasing the electrical load from 0.5 to 500 kΩ, there is a noticeable shift in resonance peak frequency and a significant increase in the magnitude of the voltage in both simulated and measured data. The changes produced by increasing the load from 100 to 500 kΩ are negligible, thus the 500 kΩ case can be considered as the open circuit condition. Regarding correlation between theory and experiment for the voltage FRFs, this is satisfactory, and the discrepancies in voltage FRF magnitudes reduce with increasing electrical load. As far as the voltage FRF resonance peak frequency is concerned, the measured short circuit and open circuit resonance peak frequencies are 46.7 Hz and 48.925 Hz, respectively. The corresponding predictions are 47.06 Hz and 49.08 Hz using DSM, and 47.3 Hz and 49.2 Hz using ANSYS. For loads of up to approximately 10 kΩ it can be observed that the (predicted/measured) output voltage for excitation at the short circuit resonance peak frequency is higher than that produced for excitation at the open circuit resonance peak frequency since the voltage resonance peak frequency is close to its short circuit condition. After exceeding this particular value of load, the voltage resonance peak frequency shifts to a value close to its open circuit condition [24]. After this switch, the output voltage magnitude is less sensitive to variation in load (as seen from Figure 10c,d, where the curves at the highest three loads are very close to each other).

**Figure 13:**
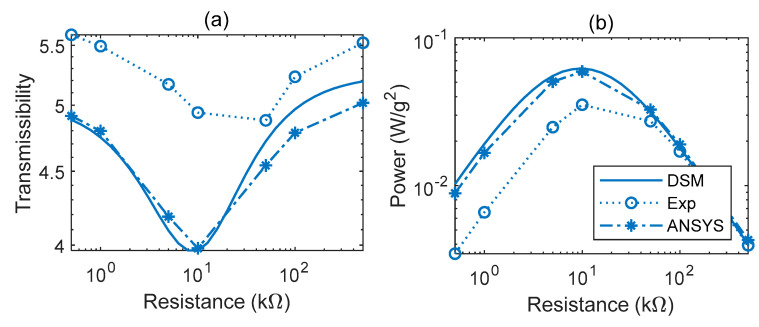
Variations of peak magnitude of the transmissibility FRF (**a**) and power FRF (**b**) with load.

With regard to the transmissibility FRFs (Figure 11a–d), the agreement between simulations and experiments is satisfactory overall. The abrupt shift in resonance peak frequency beyond ~10 kΩ observed previously in the voltage FRFs is also evident in the predicted and measured transmissibility graphs. It should be noted that the values for resonance peak frequencies predicted/observed in the voltage FRFs are slightly different from those predicted/observed in the transmissibility FRFs due to the forms of the voltage and transmissibility FRFs being different [39]. It is evident from Figure 11c,d that damping is minimal (transmissibility peak magnitude maximal) in the short circuit condition (0.5 kΩ) and open circuit condition (500 kΩ). The damping is maximal (transmissibility peak magnitude minimal) at ~10 kΩ in the predictions and between 10 and 50 kΩ in the measurement. This is in line with the estimates of the damping ratio in Table 4. It is also in line with the power FRFs (Figure 12a–d), where it is seen that the peak power dissipated is minimal in the short and open circuit conditions (0.5 kΩ and 500 kΩ, respectively), and maximal at ~10 kΩ in the predictions and between 10 and 50 kΩ in the measurement. Figure 13a,b illustrates this relation more clearly between damping and energy harvesting by showing the variation of the peak transmissibility and peak power with electrical load (more experiments are required to precisely locate the experimental resistance value for minimal peak transmissibility/maximum power dissipation).

The measured variations of the peak transmissibility and peak power with electrical load shown in Figure 13 are consistent with the corresponding predicted variations, but there are significant differences in the predicted and measured values. These are attributed to uncertainty in the various modelling parameters input into the model, e.g., uncertainty in the mechanical and electrical characteristics of THUNDER’s layer materials, manufacturing imperfections resulting in deviations from the assumed circular shape, and non-uniformity of the thickness of the layers; errors in modelling of the joints that link the THUNDER transducers to the rest of the energy harvester.

**Figure 14:**
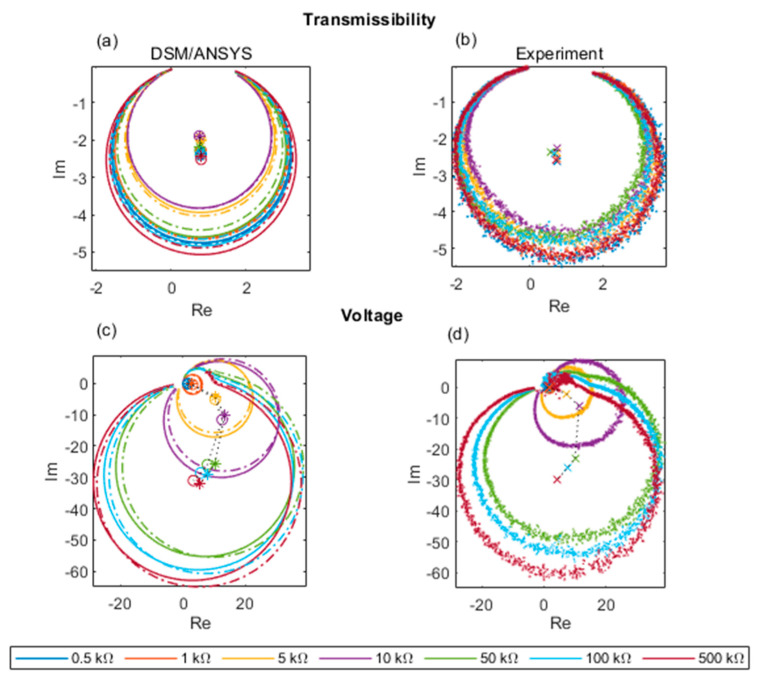
Nyquist plots of transmissibility FRF and voltage FRF for different electrical loads: (**a**) predicted transmissibility; (**b**) measured transmissibility; (**c**) predicted voltage FRF; (**d**) measured voltage FRF (dashed and solid lines in (**a**,**c**) represent data associated with ANSYS plane element model and DSM, respectively).

Energy harvesting research typically focuses on the magnitude of the FRFs, e.g., [31,36,37]. The transmissibility and voltage FRFs are complex-valued and thus have both magnitude and phase information. Rather than looking at the phase variation in isolation, a more thorough approach for validating the predicted effect of the phase variation is to plot the predicted and measured FRFs on a complex plane (imaginary part vs. real part), i.e., as Nyquist plots (as done in [24] for a simple base-excited cantilever harvester). Nyquist plots for theoretical and experimental FRF data are shown in Figure 14. The experimental transmissibility Nyquist plots in Figure 14b were used to generate the damping ratio data in the last column of Table 4 (after circle fitting). From Figure 14a,b it is clear that the transmissibility FRF continues to follow Equation (41) as the electrical load is increased, its Nyquist plot remaining approximately circular and the orientation of the circle practically unaffected by the magnitude of the load. The Nyquist plots for the predicted and measured voltage FRFs (Figure 14c,d) are also approximately circular for all electrical loads but their orientation is highly dependent on the magnitude of the electrical load. The satisfactory agreement between theoretical and experimental Nyquist plots for both transmissibility and voltage FRF provides a further source of validation for the analysis in this paper.

## 6. Conclusions

This paper has presented an analytical model of a piezoelectric energy harvesting curved beam based on the dynamic stiffness method (DSM) and applied it to predict the measured output of an energy harvester that uses a commercial curved transducer (THUNDER TH-7R). The design of the device is novel for energy harvesting purposes in that the input excitation is applied to one end of the parallel transducers in the longitudinal direction, vibrating a tip mass attached at the other end. The DSM modelled the transducer as a curved laminated beam, with no limitations on the degree of initial curvature, and including all layers of the transducer. As a secondary novel contribution, the same device was also modelled using commercial FE software (ANSYS) through alternative element-type models. The results of DSM were consistent with those of ANSYS. Predictions by both ANSYS and DSM for the transmissibility and voltage FRFs at various electrical loads ranging from short circuit to open circuit showed a satisfactory degree of correlation with the experimental results in all aspects: magnitude, phase, Nyquist plots, and resonance frequency shift. The range of electrical load for maximal power generation and maximal damping was correctly identified. The shift in resonance frequency while changing the electrical load from short circuit to open circuit was predicted to be 4.01% by ANSYS and 4.3% by DSM, which compared well with the shift of 4.7% observed from the experimental data.

It is concluded that the presented dynamic stiffness model is sufficiently accurate for commercial curved transducers used in applications where the predominate mode of vibration is beam-like. In such situations, it can be used in preference to FE software with less computational effort due to the drastically reduced number of elements required and the ease of application on general purpose coding software, such as MATLAB. Moreover, the matrix assembly procedure presented allows application to more complex transducers comprising segments of different radius of curvature.

## Figures and Tables

**Table 1:** Properties used for THUNDER [32].

Layer No. (*k*)	Material	Elastic Modulus, Y (GPa)	$\mathrm{Density},\;\rho \;\left(\mathrm{kg}\;m^{-3}\right)$	$\mathrm{Piezoelectric}\;\mathrm{Stress}\;\mathrm{Constant},\;e_{31}\;\left(C\;m^{-2}\right)$	$\mathrm{Permittivity}\;(\mathrm{At}\;\mathrm{Constant}\;\mathrm{Stress}),\;\epsilon_{33}^{T}\;\left(nF\;m^{-1}\right)$	Thickness, h (μm)	Poisson Ratio (For FE Models)
1	Steel	193	8000	-	-	203	0.25
3	PZT	67	7600	−12.73	16.82	254	0.31
5	Aluminum	70	2700	-	-	25.4	0.33
2, 4	Adhesive	3.45	2200	-	-	25.4	0.40

**Table 2:** Dimensions used for THUNDER (all properties are given in mm).

Property	Value
Dome height, ***H***	9.55
Overall Length, ***L***	97.66
Width, ***b***	73.41
Active length	72.3

**Table 3:** Natural frequencies (Hz) of test rig obtained with ANSYS for pure short circuited load condition (no electrical effect), together with corresponding DSM results.

Circumferential Mode Number	Natural Frequency: ANSYS Plane Elements (Hz)	Natural Frequency: ANSYS Shell Elements (Hz)	Natural Frequency: ANSYS Solid Elements (Hz)	% Difference (Plane vs. Shell)	% Difference (Plane vs. Solid)	Natural Frequency: DSM (Hz)	% Difference (Plane vs. DSM)
1	47.28	48.80	49.73	−3.1	−4.9	47.0	0.6
2	233.74	246.15	246.63	−5.0	−5.2	237.0	−1.4
3	388.32	408.56	409.51	−5.0	−5.2	395.2	−1.8
4	785.68	811.63	826.02	−3.2	−4.9	783.4	0.3

**Table 4:** Estimates of damping ratio from transmissibility FRF (experiment) using three different methods.

Load (kΩ)	Damping Ratio (%)		
	Resonance Point Method, Equations (38a) and (38b)	Half-Power Point Method, Equations (39)–(41)	Circle-Fit Method, Equation (41) and Figure 7
0.5	9.07	8.81	8.91
1	9.22	9.28	8.85
5	10.10	9.71	9.33
10	10.54	10.20	9.90
50	10.22	9.59	10.24
100	9.89	8.99	9.15
500	9.23	8.75	8.48

## Data Availability

The data presented in this study can be made available upon request to the corresponding author.

## Appendix A

The stress and stress-moment resultants obtained from Equation (2) yield the following expressions:

(A1a) $$N=\vartheta_{N}v+\left(\frac{A}{r}-B\frac{\partial^{2}}{\partial s^{2}}\right)w+\left(A+\frac{B}{r}\right)\frac{\partial }{\partial s}u+\left(\frac{\bar{A}}{r}-\bar{B}\frac{\partial^{2}}{\partial s^{2}}\right)\dot{w}+\left(\bar{A}+\frac{\bar{B}}{r}\right)\frac{\partial }{\partial s}\dot{u}$$

(A1b) $$M=\vartheta_{M}v+\left(\frac{B}{r}-D\frac{\partial^{2}}{\partial s^{2}}\right)w+\left(B+\frac{D}{r}\right)\frac{\partial }{\partial s}u+\left(\frac{\bar{B}}{r}-\bar{D}\frac{\partial^{2}}{\partial s^{2}}\right)\dot{w}+\left(\bar{B}+\frac{\bar{D}}{r}\right)\frac{\partial }{\partial s}\dot{u}$$

where:

N and M are the internal axial load and bending moment, respectively, shown in Figure 2 along with the shear force V=∂M/∂s. The remaining symbols in Equation (A1) are defined as follows:

(A2a) $$A=b\overset{n}{\underset{k=1}{\sum }}Y_{k}h_{k},$$

(A2b) $$B=b\overset{n}{\underset{k=1}{\sum }}Y_{k}h_{k}{\bar{z}}_{k},$$

(A2c) $$D=\frac{1}{3}b\overset{n}{\underset{k=1}{\sum }}Y_{k}\left(z_{k}{}^{3}-z_{k-1}{}^{3}\right)$$

(A2d) $$\bar{A}=b\overset{n}{\underset{k=1}{\sum }}c_{k}h_{k},$$

(A2e) $$\bar{B}=b\overset{n}{\underset{k=1}{\sum }}c_{k}h_{k}{\bar{z}}_{k},$$

(A2f) $$\bar{D}=\frac{1}{3}b\overset{n}{\underset{k=1}{\sum }}c_{k}\left(z_{k}{}^{3}-z_{k-1}{}^{3}\right)$$

(A2g) $$\vartheta_{N}=be_{31},$$

(A2h) $$\vartheta_{M}=be_{31}z_{pc},\;\;$$

(A2i) $$z_{pc}={\bar{z}}_{p}$$

(A2j) $$z_{k}=z_{k-1}+h_{k},\;\;$$

(A2k) $${\bar{z}}_{k}=\frac{1}{2}\left(z_{k}+z_{k-1}\right)$$

In Equation (A2) above, $z_{k}$ is the *z*-coordinate (from the mid-surface of the segment) of the upper surface of layer no. *k*, and $z_{0}$ is the *z*-coordinate of the lower surface of layer no. 1.

The equations of motion for the circular laminated beam segment without externally applied forces along its length (between its ends) are given as [46]:

(A3a) $$\frac{N}{r}-\frac{\partial^{2}M}{\partial s^{2}}+m\ddot{w}+c_{a}\dot{w}=0$$

(A3b) $$-\frac{\partial N}{\partial s}-\frac{1}{r}\frac{\partial M}{\partial s}+m\ddot{u}+c_{a}\dot{u}=0$$

Placing Equation (A2) into Equation (A3) leads to

(A4a) $$K_{w}w+Ku+m\ddot{w}+\left({\bar{K}}_{w}+c_{a}\right)\dot{w}+\bar{K}\dot{u}+\chi_{w}v=0$$

(A4b) $$-Kw+K_{u}u+m\ddot{u}-\bar{K}\dot{w}+\left({\bar{K}}_{u}+c_{a}\right)\dot{u}+\chi_{u}v=0$$

where

(A5a) $$K_{w}=D\frac{\partial^{4}}{\partial s^{4}}-\frac{2B}{r}\frac{\partial^{2}}{\partial s^{2}}+\frac{A}{r^{2}},$$

(A5b) $${\bar{K}}_{w}=\bar{D}\frac{\partial^{4}}{\partial s^{4}}-\frac{2\bar{B}}{r}\frac{\partial^{2}}{\partial s^{2}}+\frac{\bar{A}}{r^{2}}$$

(A5c) $$K=-\left(B+\frac{D}{r}\right)\frac{\partial^{3}}{\partial s^{3}}+\frac{1}{r}\left(A+\frac{B}{r}\right)\frac{\partial }{\partial s},$$

(A5d) $$\bar{K}=-\left(\bar{B}+\frac{\bar{D}}{r}\right)\frac{\partial^{3}}{\partial s^{3}}+\frac{1}{r}\left(\bar{A}+\frac{\bar{B}}{r}\right)\frac{\partial }{\partial s}$$

(A5e) $$K_{u}=-\left(A+\frac{2B}{r}+\frac{D}{r^{2}}\right)\frac{\partial^{2}}{\partial s^{2}},$$

(A5f) $${\bar{K}}_{u}=-\left(\bar{A}+\frac{2\bar{B}}{r}+\frac{\bar{D}}{r^{2}}\right)\frac{\partial^{2}}{\partial s^{2}}$$

(A5g) $$\chi_{w}=\frac{\vartheta_{N}}{r}-\vartheta_{M}\frac{\partial^{2}}{\partial s^{2}},$$

(A5h) $$\chi_{u}=-\left(\vartheta_{N}+\frac{\vartheta_{M}}{r}\right)\frac{\partial }{\partial s}$$

(A5i) $$m=b\overset{n}{\underset{k=1}{\sum }}\rho_{k}h_{k}$$
